# A novel in vitro model of metastasis supporting passive shedding hypothesis from murine pancreatic cancer Panc-02

**DOI:** 10.1007/s10616-019-00341-2

**Published:** 2019-09-09

**Authors:** M. P. Krzykawski, R. Krzykawska, M. Paw, J. Czyz, J. Marcinkiewicz

**Affiliations:** 1 Department of Immunology, Jagiellonian University Medical College, Kraków, Poland; 2 Department of Cell Biology, Faculty of Biochemistry, Biophysics and Biotechnology, Jagiellonian University, Kraków, Poland

**Keywords:** passive sheding mechanism, Panc-02, Pancreatic cancer metastasis, Cancer model, In vitro metastatic selection

## Abstract

Cancer metastasis is believed to happen through active intravasation but there might be also another way to metastasize. According to passive shedding hypothesis, proposed by Munn et al., tumor cells detach from the tumor mass and passively shed to blood stream through leaky blood vessels. We propose a novel In Vitro Migrational Selection (IVMS) assay that enables the pre-selection of invasive pancreatic cancer Panc-02 cells and create a model of passive shedding. We established invasive sub-cell line of murine pancreatic cancer Panc-02 cells (refered to as Panc02-RS), which exhibited higher metastatic potential in vivo and at the same time decrease in vitro migratory skills, comparing to the initial Panc-02 cell line. In in vitro cell cultures Panc-02 spontaneously detached from the cell culture surface and later reattached and colonized new areas. We believe it can mimic the new way of metastasis, namely passive shedding. We concentrated on Panc-02 model but believe that IVMS might be used to create sub cell lines of many solid tumors to model passive shedding. Our results support the passive shedding hypothesis.

## Introduction

Lethality of pancreatic cancer is determined by its metastases, however the mechanism(s) that governs the metastatic cascade of pancreatic cancer are still obscure (Vanharanta and Massagué 2013). This process is triggered and maintained by complex intercellular communication systems, constituted by humoral factors, intercellular exchange of mechanical stimuli and gap junctional intercellular communication (Feygenzon et al. 2017). Cells evoke secular and permanent phenotypic shifts, such as EMT (epithelial-mezenchymal transition) that increase the predilection of cancer cells to colonize the distant organs. Cells that undergo EMT seem to be preparing for migration: they change to more spindle shaped morphology, produce more ECM degrading enzymes and increase the resistance to apoptosis (Kalluri and Neilson 2003). On the molecular level EMT is primarily described by loss of E-cadherin, a protein responsible for cell–cell adhesion (Ramis-Conde et al. 2009). Equally important is N-cadherin upregulation. N-cadherin provides an important skill for metastatic cells (Nakajima et al. 2004). After reaching a narrow blood vessel a metastatic cell can attach to the blood vessel wall where N-cadherin undergo phosphorylation necessary for adjacent endothelial cells and allow the cancer cell to enter the tissue (Ramis-Conde et al. 2009). Precise elucidation of the mechanisms that determine pancreatic cancer progression depends on the establishment of experimental models that would enable the establishment and phenotypic characterization of invasive cells in strictly controlled conditions.

Cancer metastasis is a multi step process. In our study we concentrate only on the initial steps when cancer cell needs to find a way to leave the primary tumor. Today metastasis is mostly understood as a process of active migration (Keleg et al. 2003). By active migration we understand all sorts of active chemotactic attraction of a cancer cell towards more oxygenated and nourished region of the tumor closer to blood vessels. In contrast to active migration, passive shedding is a process in which cells detach from the startum and other cells and can remain in suspension for a period of time. If these suspended cells will be pushed out out of the tumor by body fluids they will reach the blood steam and migrate outside of the tumor, see Fig. 10. We want to show evidence supporting the hypothesis of passive metastasis and propose a new research model. As discussed by Munn et al. active migration is important in later extravasation stages but passive intravasation may be more important at the beginning of the metastatic process (Bockhorn et al. 2007). Butler and Gullino as well as Liotta et al. showed that millions of potentially metastatic cells are shed from the tumor every day (Liotta et al. 1976; Butler and Gullino 1975). It has not been well studied because there were no models allowing researchers to investigate this type of metastasis. We propose a method to research this mechanism and call it IVMS (in vitro metastatic selection).

Generation of metastatic cancer cell subpopulations in vitro is based on microdissection and repeated micro-invasion assay, where cancer cell populations are enriched with invasive cells by the collection-reseed cycles or Matrigel- and/or endothelium-coated microporeous membranes (Albini 2016; Liu 2010). These approaches are based on active adhesion-dependent mechanism of cancer cell invasion (Bruns et al. 1999; Xie et al. 2001; Shi et al. 1999). For example, repeated injection of COLO 357 cells into a pancreas resulted in the propagation of malignant cell lineage (Bruns et al. 1999). Application of in vitro models eliminates the bias between syngenic and xenogenic models because immune system plays an essential role in selecting cells with acceptable immunity of the surface markers.

To establish metastatic cancer cell populations in vivo, one has to repeat transfer of metastatic cells between animals. Xiangdong et al. produced a cell sub-line Panc02-H7 characterized by higher metastatic potential than a wild type Panc-02 cells. The drawback of most common in vivo approaches towards pre-selection of invasive cells is the pressure of immune system (Xie et al. 2001).

Our results support the concept of passive shedding. Theoretical concept proposed by Munn et al. has been omitted due to lack of evidence supporting that idea and methods necessary to investigate it (Bockhorn et al. 2007). In this work we want to revive that concept by adding a research method necessary for research as well as by adding direct arguments that such a process an important metastatic factor. IVMS is a new method to create new models that can shed new light on the passive shedding mechanism.

## Materials and methods

### Cell line and cell culture

Murine pancreatic adenocarcinoma Panc-02 cell line has been purchased from DTP, DCTD Tumor Repository, originally isolated by Corbett et al. (1984) from C57BL/6 murine tumor. Cells were cultured in RPMI (Lonza, Basel, Switzerland) supplemented with 10% FBS (Euro Clone) at standard conditions.

### IVMS—in vitro migrational selection assay

We describe one cycle of metastatic selection. Every stage of the procedure will be repeated many times. To achieve the full selection it is necessary to repeat this process until an expected spindle-shape phenotype is obtained. In order to obtain more spindle shape cell line with the increased number of freely floating cells it is advised to repeat the procedure 12–14 times. As a result, Panc02-RS invasive population contained both suspended and adherent cell sub-populations. For IVMS assay 75 cm^2^ cell culture flasks were used.

**Fig. 1:**
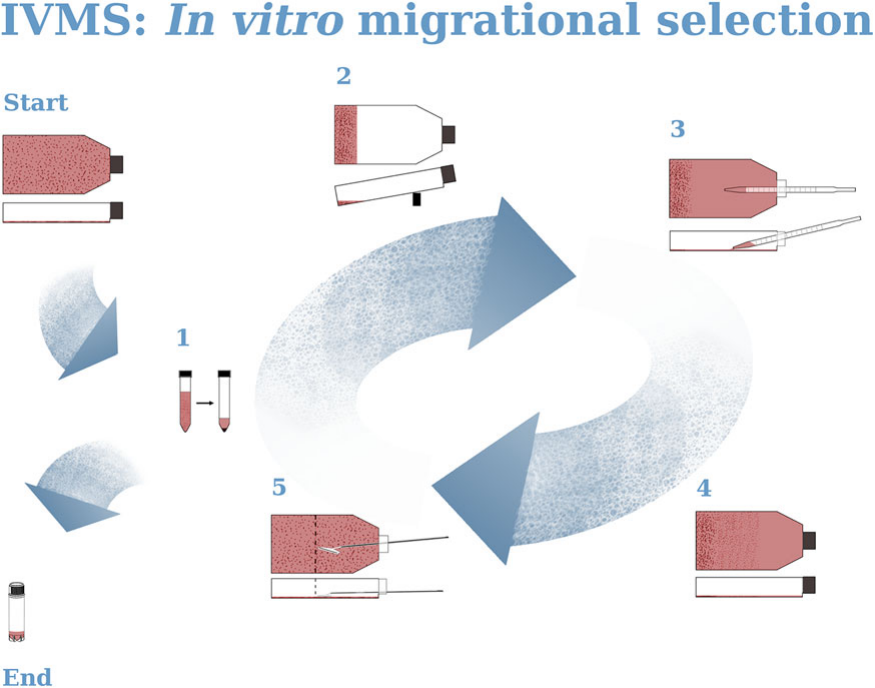
Schematic visualization of the IVMS (*In Vitro* Metastatic Selection) procedure. IVMS is a procedure developed to preselect metastatic cells in vitro. “Start” stage begins with any adhesive cell culture and in stage “1” enters the cycle. Cycle can be repeated many times to obtain expected result for example higher number of cells in suspension. Stage”1” is also a moment to exit the cycle and prepare banks for further research or continue directly with experiments. See text for the details of the procedure

The “Start” stage of IVMS begins with cells growing equally allover the flask, with 90–95% confluence. Collect the cell culture media and preserve it. Add 3 ml of cell culture media to the flask, mechanically remove cells (with cell scraper) and collect the cells into the used cell culture media. It is recommended to split cells in half, freeze one portion for later, to use it as reference. Use the second half for IVMS in Stage 1. shown in Fig. 1 Start .

Stage 1: Centrifuge cells for 10 min, at 1000RPM to obtain a pellet. Remove the supernatant and add 2 ml of fresh culture media. Shown in Fig. 1(1)

Stage 2: Pipette cells with fresh media to re-suspend the pellet and pour cells into a new cell culture flask. Keep the new cell culture flask on the back side (as shown in Fig. 1(2)) and pour the cells suspension precisely at the end of the flask, so that cells could grow only on one side of the bottle. Remember to keep the flask tilted to same a degree. It is necessary not to drop the cells in any other place than the back end of the flask and keep it all the time on a slope. Place it in the incubator keeping it on the slope for 24 h.

Stage 3: After 24 h remove medium from the end side of the flask. Flask should be kept in leaning position. Cells should be attached to the cell culture flask only at one side of the bottle as shown in Fig. 1(3). Add 10 ml of culture media and place the flask back into the incubator, let it lay flat. You should see full confluence of growing cells at the side of the flask and no cells should be growing at the area close to the cap.

Stage 4: Within 3–5 days you will start observing cells showing up on the cap side as shown in Fig. 1(4).

Stage 5: The day when you will see cells with confluence of about 80–90% at the cap side you should mechanically remove cells from half of the bottle, normally it took about 3 days to grow cells allover the bottle, see Fig. 1(5). When repeating this process remember to scrape the cells from the cap side of the bottle where no cells were seeded at the beginning. If you wish to continue the IVMS selection repeat the process from stage 1 and use freshly scraped cells. If you have not obtained expected results yet but you are interested in the mechanism behind the process you are researching at this stage you may also collect the second half of cells and preserve by banking.

If expected results have been obtained, stage 5 is the moment to stop the procedure and turn to the “End” stage.

Stage “End”: Collect the cells from the entire bottle and centrifuge. Preserve cells by deep freezing for further research.

Additional information: to obtain more information, stage 4 can be done in a fixed number of days later at stage 5 a cell count of the scraped cells and the suspended cells will show an increase in numbers.

### In vivo metastatic assay

C57BL/6 is a mice purchased from Jackson Laboratory (Bar Harbor, Maine, USA). Male mice used in this study were 8–12 week old, housed at controlled condition (21 °C; 12 h/12 h dark/light cycle) and had free access to food and water. Procedures approved by the University’s Animal Ethic Committee (Decision No: 140/2015; 94/2014). For the analyses of Panc-02: Panc-02 and Panc02-RS metastatic potential, 0.5 × 10^3^ cells in 100 ul aqueous suspension (Cell Culture Grade) (Krzykawski et al. 2015) were implanted on the back s.c. The total number of 18 mice were used in this experiment, 6 mice for Panc-02 cell line and 12 mice for Panc02-RS cell line. Volumetric measurements of primary tumor size (from three diameters) were made using caliper. Mice were mildly anesthetized by inhaled isoflurane for 20 s. Tumor growth was measured every week for 7 weeks. Mice were euthanized by cervical dislocation after isofurane inhalation for 60 s. Metastatic potential of Panc-02 and Panc02-RS sub-populations was measured by the number of the metastatic tumor lesions in various organs.

### Invasive potential in vitro

For the time-lapse videomicroscopic analyses of cell migration, the cells were plated into the 24-well culture plates. After 24 or 48 h, cell movement was recorded with a Leica DMI6000B time-lapse system equipped with a temperature/CO_2_ chamber, IMC contrast optics and a cooled, digital DFC360FX CCD camera. The cell trajectories were constructed from a sequence of cell centroid positions recorded at 420 s time intervals using a dry 20× , NA-0.75 objective. Total length of cell trajectory (μm), total length of cell displacement (i.e. the distance from the starting point directly to the cell’s final position; μm), average speed of cell movement, i.e. the total length of cell trajectory/time of recording (μm/min) and the average rate of cell displacement, i.e. the distance from the starting point directly to the cell’s final position/time of recording (μm/min) were quantified from the trajectories of > 60 cells per each condition with the Hiro program (written by W. Czapla).

### Cell transmigrational potential

Cells were seeded on the transwell inserts in 24-well plates at 10,000 cells/well. After 24 h of cultures, the insert was transferred to another well for the next 24 h. This step was repeated five times and cells were counted. The results were shown as the ratio of cell numbers per well to the number of cells in referrence well taking into account the cell proliferation time.

### Cell motility analysis

Circular diagrams show single cell trajectories starting with the initial point at the origin of the plot registered for 6 h or 8 h; N > 50). Results are representative of three independent experiments *p < 0.05 (tested with Annova, followed by Bonferroni test). Square graphs show a correlation of a total length of trajectory compared to displacement. It shows how much of the cell movement is directed and how much is just a random walk. Low Density (LD) = 25% confluence; High Density (HD) = 90% confluence; Average speed [um/min]; Average displacement [um], *p < 0.05.

For the cell motility of Panc-02 and Panc02-RS cells in High Density (HG) and Low Density (LD) cells were seeded with the density of 5 × 10^3^ and 30 × 10^3^ cells in LD and HD respectively (Fig. 6). Cells were cultured for 24 h before the experiment. Cells were measured for about 6 h, 50 frames, each frame every 7 min. Each measurement included 60 cells.

For the cell motility of Panc-02 and Panc02-RS cell lines across different selection cycles (before the selection, after 5 selection cycles, after 9 selection cycles, after the entire selection—12 cycles) cells were seeded on a 12 well plate, 4 × 10^3^ cells/cm^2^ (Fig. 7). Cells were cultured for about 8 h and observed with 70 frames, 1 frame every 7 min. Each measurement included 60 cells.

### Cell proliferation analyses

Cells were seeded at a density of 3000 cells per well into the 48-well plates and cultured with RPMI supplemented with 10% FBS for 9 days. Every 24 h, adherent and suspended cells were collected and counted in triplicates from 3 different wells. Percentage of living cells were determined by trypan blue staining.

### Conditioned media experiment and preparation of conditioned mediated

Conditioned media from Panc-02 and Panc02-RS cell were prepared. Cells were seeded in a 75 cm^2^ culture flask at full density. After 3 days cell culture media were collected, centrifuged to remove cells and supernatant (conditioned media) was frozen.

For the analyses of the effect of cell secretome on cell motility, cells were seeded at a density of 5000 cells per cm^2^ in a standard culture medium in a 12-well plates. After 24 h medium were replaced on the mixture of fresh and conditioned culture medium at the ratio 1:3, 1:1 and 3:1. After 24 h of stimulation, non-adherent cells in each well were counted.

### Gap junctional intercellular coupling (GIJC)

Cells were seeded at a density of 30,000/cm^2^. After 24 h, calcein/DiI-loaded donor cells (Life Technologies Corp.) were plated on the monolayers of acceptor cells grown in Petri dishes at a density of 20 000 per dish and the number of donors, calceine-transferred cells and acceptors was measured by the BD LSR Fortessa X-20 flow cytometer (BD Biosciences) and the results of gap junctional coupling were expressed as the ratio of acceptor cell numbers to donor cell numbers. Statistical analysis has been done with t-Student test.

### Graphics

All graphical work has been done in GIMP 2.8.10.

### Statistical analyses

Values was shown as mean ± SEM. Statistical significance of the differences was estimated with the t-student test (*p < 0.05), in vivo tumor growth were tested with one-way ANOVA.

## Results and discussion

**Fig. 2:**
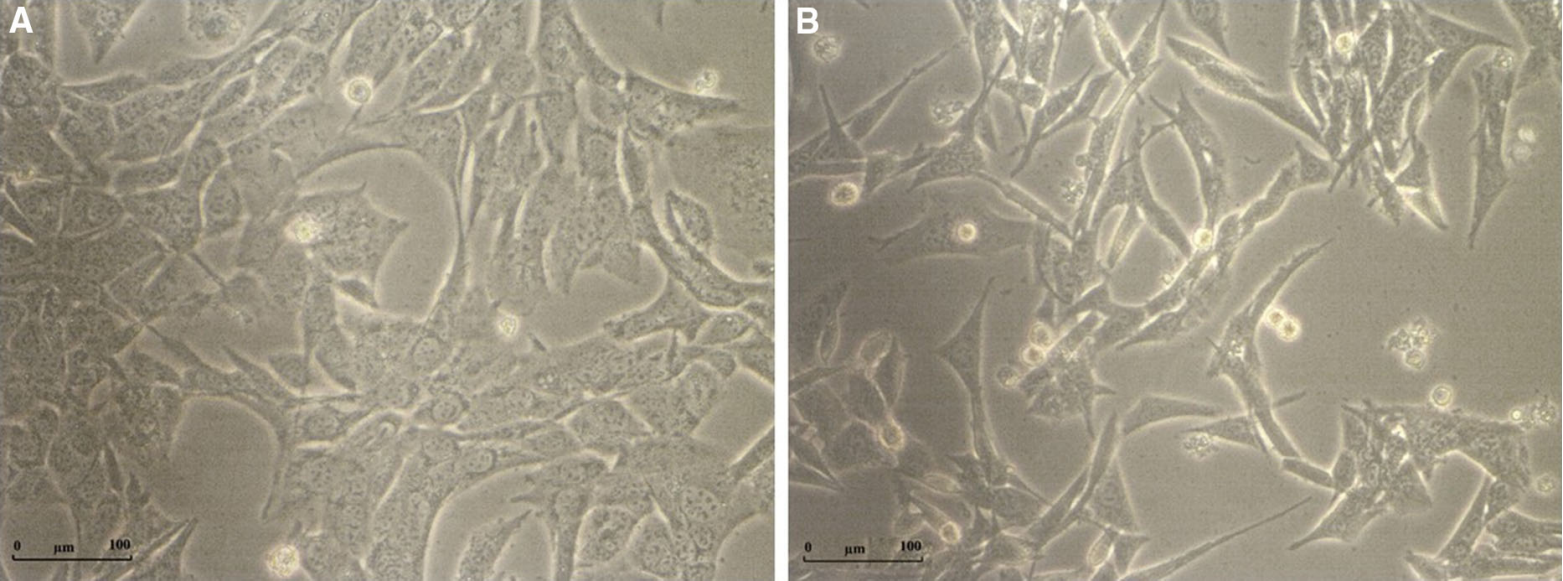
Morphological heterogeneity of cells before (Panc-02) and after IVMS (Panc02-RS). **a** Panc-02. **b** Panc02-RS. Panc02-RS cells underwent IVMS, resemble more spindle shaped cells and are less attached to the surface comparing to Panc-02

IVMS enabled us to establish a subpopulation of Panc02-RS cells, which in contrast to their flattened and strongly attached Panc-02 counterparts, were more of a spindle shape-type and less attached to the substrate (Fig. 2). Such cellular shape is characteristic for the cells constituting the invasive front of cancer.

IVMS assay was at the beginning designed to select faster migrating cells. We assumed that cells will migrate on the surface to the empty end of the culture flask (shown in Fig. 1—IVMS assay). Within three days we already observed some cells on the cap side of the flask. It would mean that cells could migrate at a speed of about 3 cm per day what is impossible. Cells can migrate with a speed of up to few micrometers per minute (Baumann 2015). It meant that cells migrated another way. We observed some cells in suspension of Panc-02 cell culture, normally those suspended cells are described as dead or apoptotic. To test cells suspended in the culture media we took the culture media with suspended cells and poured it into a new flask. Next day we found some cells growing in the new flask. It meant that some of the living cells had been at least partially suspended in the culture media. It explained the way how these cells migrated so quickly during the IVMS assay. IVMS is built out of repeatable cycles and after about 10–12 cycles we could observe a growing number of cells in suspension (data not shown). Apparently, these cells are able to transiently detach from the substratum and to colonize the empty spaces of the flask. Later we have also observed higher number of Panc02-RS cells in suspension.

**Fig. 3:**
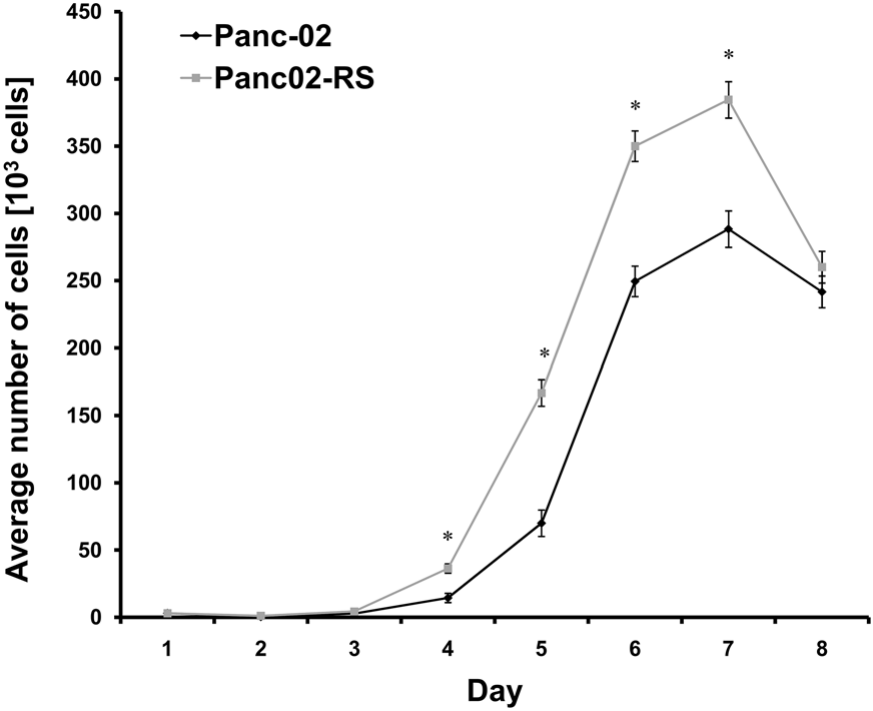
Comparison of growth between Panc-02 and Panc02-RS. Cells that underwent IVMS grew faster and reached a higher number of cells per well and higher cell culture density,* p < 0.05

As shown in Fig. 3 Panc02-RS cells started dividing earlier what may be an argument supporting the idea that Panc02-RS cells have an ease to accommodate to new environment. We did not observe accelerated proliferation of Panc02-RS cells. These cells however were more efficient to recover from the lag phase after seeding (Fig. 3), grew to a higher total number of cells probably due to high number of living cells in suspension.

### Next we wanted to find out if this in vitro process could influence the in vivo migration/metastasis?

**Fig. 4:**
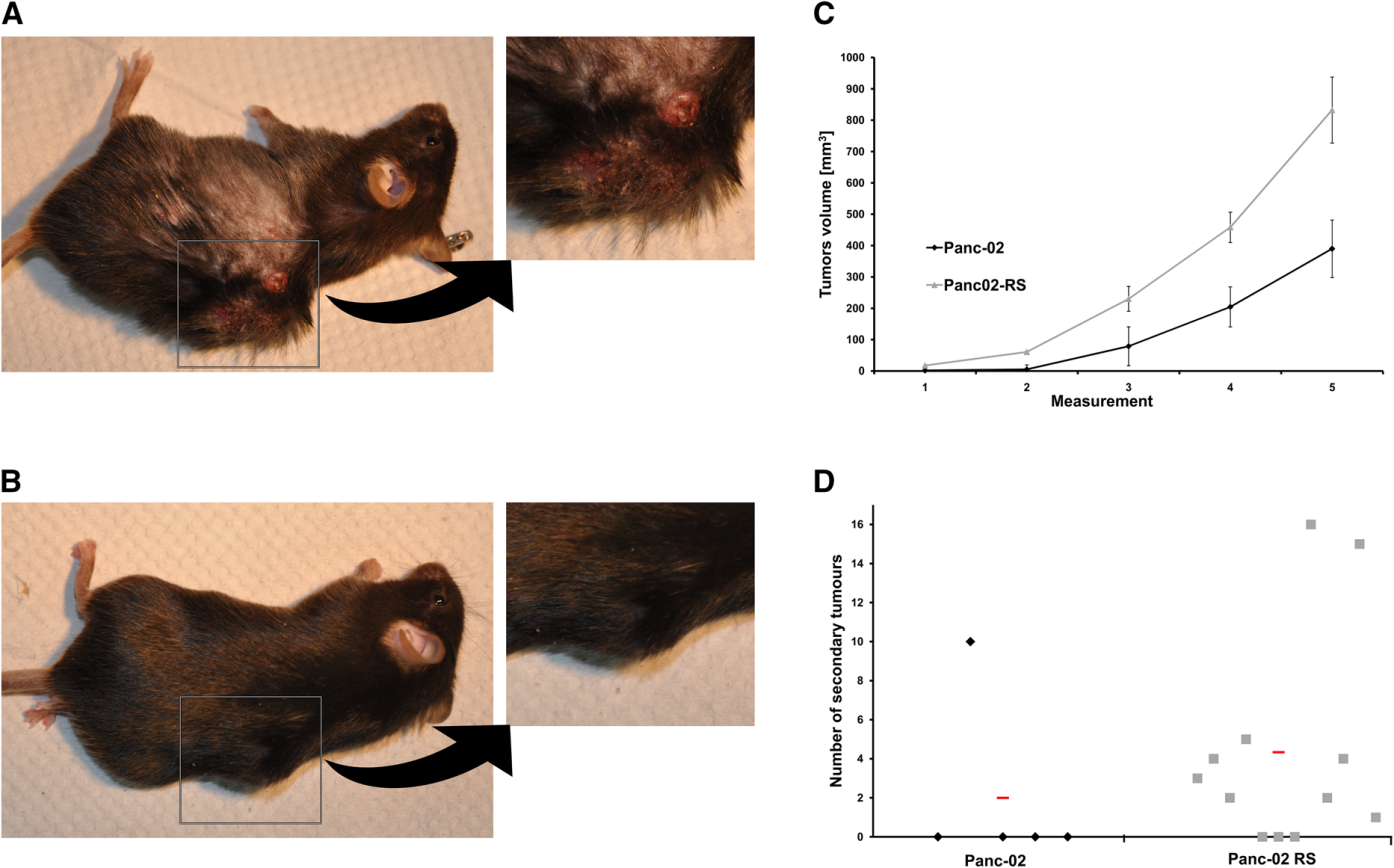
*In vivo* tumor growth and metastasis of Panc-02 and Panc02-RS cell lines. **a** Mouse bearing Panc-02RS tumor. **b** Mouse bearing Panc02 tumor. **c** Comparisons of tumors from Panc-02 and Panc02-RS cell lines, p < 0.05. **d** Number of metastases (visible by eye) combined from all organs mainly lungs, analyzed 7 weeks after injection

The invasive morphology of Panc02-RS cells was correlated with their metastatic potential in vivo. Figure 4D clearly shows that Panc02-RS cells tend to generate more metastases than their wild type counterparts. Wild-type Panc-02 cells did not produce metastases in mice (except one animal), whereas 9 out of 12 mice injected with Panc02-RS cells developed macroscopic metastases. These data show that IVMS procedure enables the isolation of invasive sub-population(s) of Panc02-RS cells. Panc02-RS established a detectable tumor sooner and kept growing faster throughout the experiment (Fig. 4c).

In Fig. 4c one can see the comparison of growth between Panc-02 and Panc02-RS tumors. Panc02-RS cells started dividing earlier and produced larger tumors than Panc-02. Figure 4d shows that Panc02-RS cells were also more metastatic in the same time period. Note that spindle shape of Panc02-RS cells (Fig. 2b) correlates with their invasive potential in vivo.

While working with the animal models we have made further observations and interestingly for the further research we have also seen that mice carrying Panc02-RS tumors had locally thinned fur (see Fig. 4a, b). Also the visual appearance of Panc02-RS derived tumors has been more gross. We have not measured this observation.

### Did we select a more aggressive metastatic Panc-02 sub-cell line?

**Fig. 5:**
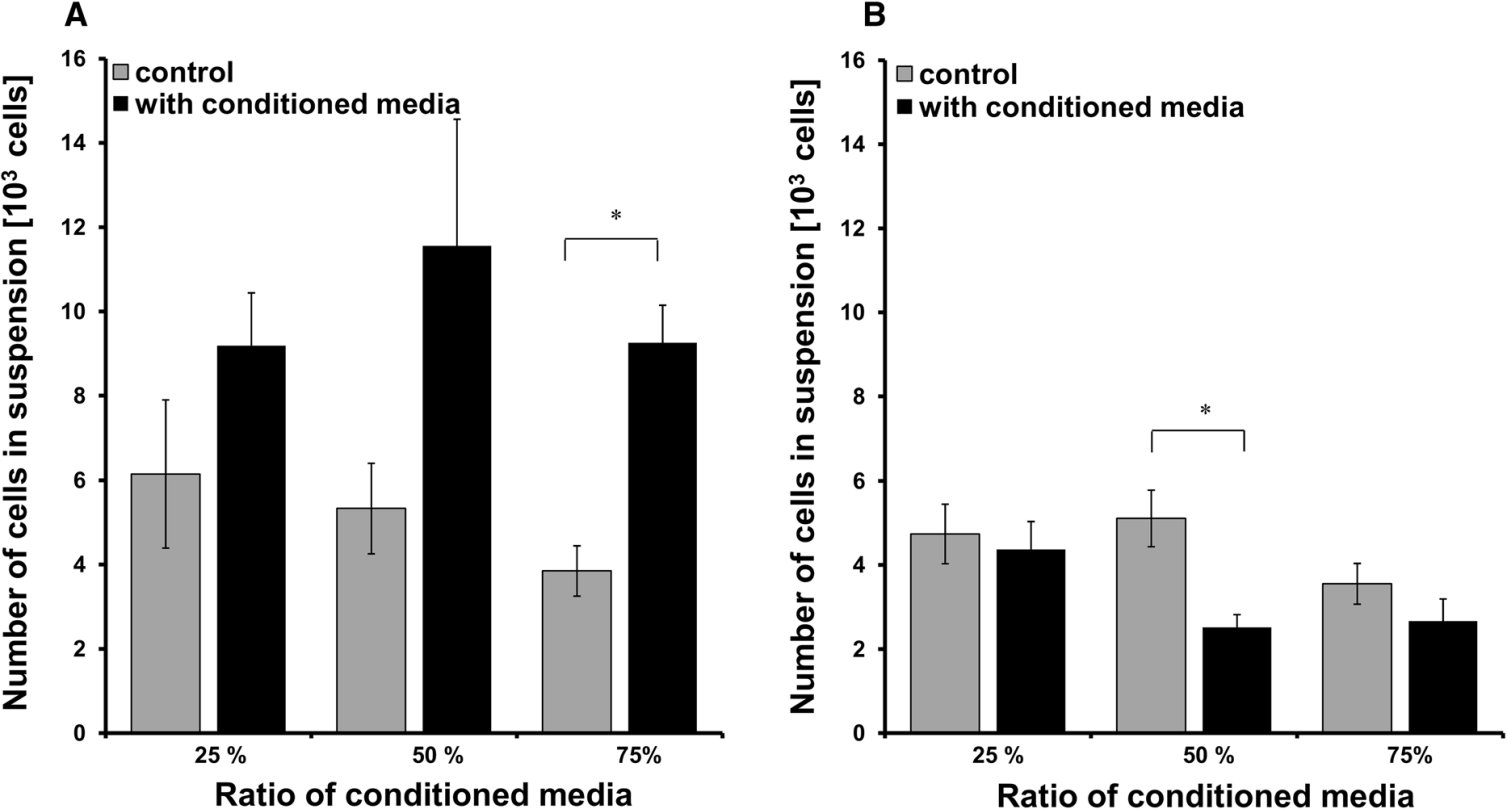
Panc-02 and Panc02-RS cells stimulation with conditioned media. **a** Panc-02 cells were stimulated with culture media conditioned by culturing in Panc02-RS cell, compared to control after 24 h, *p < 0.05. **b** Panc02-RS cells were stimulated with culture media conditioned by Panc-02 cells, compared to control after 24 h, *p < 0.05

We wanted to find out if the observed differences in numbers of suspended cells between Panc-02 and Panc02-RS come from some intracellular mechanism or from cell culture media. We added media from Panc02-RS to Panc-02 in three proportions: 25%, 50% and 75% and found an increasing number of cells suspended in cell culture media what is a hallmark of Panc02-RS cell and connects it to EMT (Fig. 5a). Similarly the addition of the same proportions of media from Panc-02 to Panc02-RS decreased the number of freely floating cells (Fig. 5b).

These data indicate the existence of paracrine loops established between Panc-02 cell sub-populations. Panc02-RS cells release humoral factors that stimulate phenotypic shifts of Panc-02 cells towards a more non-adherent phenotype. Although, these hypothetical factors remain to be identified, they induce phenotypic shifts in Panc-02 cells that transiently attenuate their adhesiveness and facilitate their passive movement across the invasive front of Panc-02 cells during IVMS assay.

As an alternative to IVMS one can make several clones and testing for higher metastatic potential. It is often seen as an option but we assumed that there are also inter-cellular processes that influence metastasis and wanted to maintain as much of the cellular physiology. We believe that the balance and cell communication play a crucial role in the physiology of metastasis. As shown in Fig. 5, the observed process is not based on single mutated cell phenotype but rather on dynamic interactions of humoral factors. Details of this process are yet to be uncovered.

### Ok, so we have a cell line that grows faster as a tumor and produces more metastasis but how it happenes?

Is it only by detachment from the surface or is it for example due to faster active migration? To answer this question we have measured the speed of migration of Panc-02 and Panc02-RS cells.

**Fig. 6:**
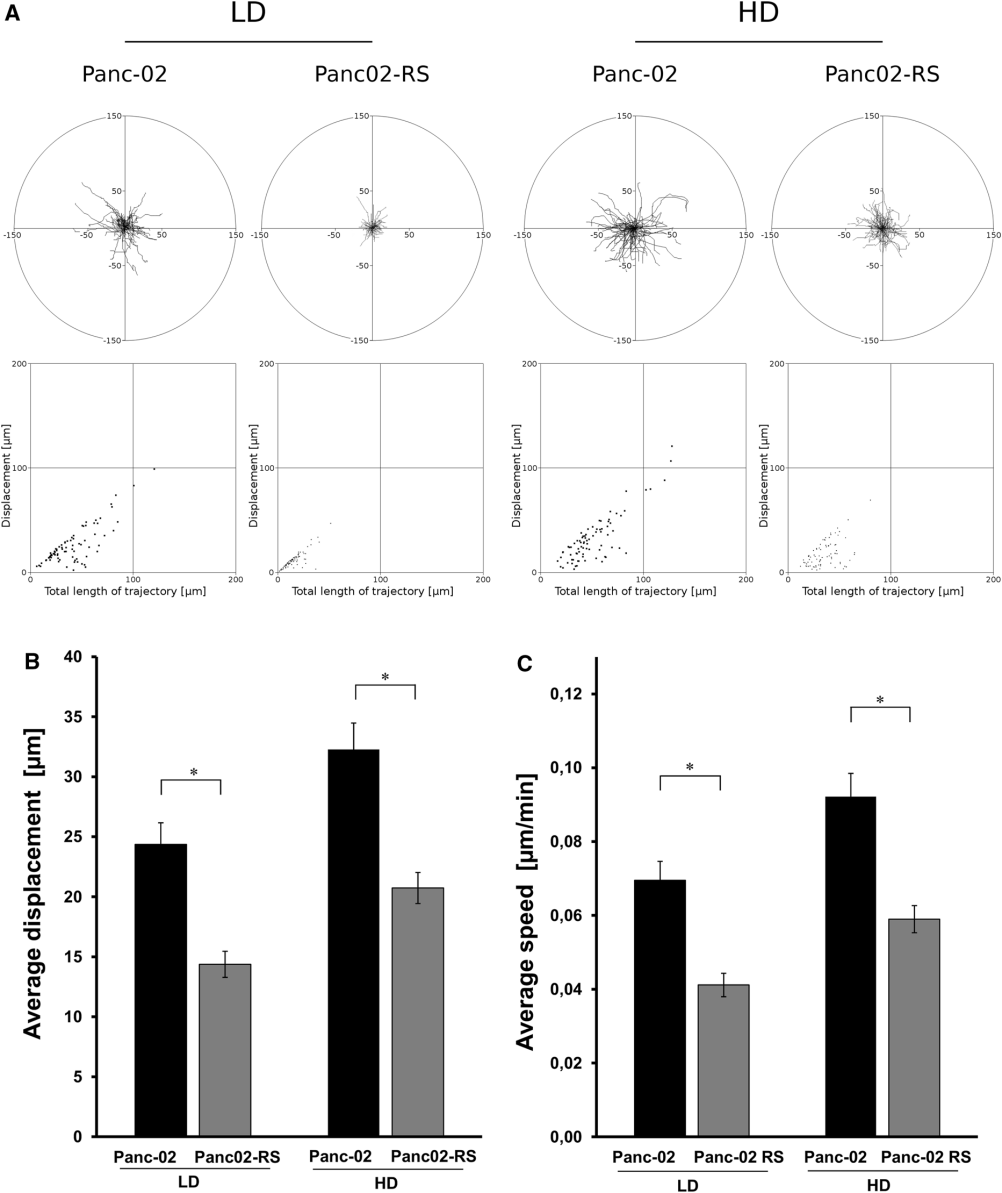
Density dependent cell motility of Panc-02 and Panc02-RS cell lines; **a** 6 h time lapse video microscopy observation at the LD (Low Density) and HD (High Density). **b** Graph showing a displacement comparing Panc-02 and Panc02-RS cell lines in LD and HG, * p < 0.05. **c** Graph C is showing a speed of migration comparing Panc-02 to Panc02-RS cell lines in LD and HG,* p < 0.05

We expected to see an increase in migration potential of Panc02-RS comparing to the Panc-02 cells because were preselected to become more metastatic what was shown by in in vivo experiments. Surprisingly Panc-02 migration has been decreased in tests like total cell displacement and speed of migration. Panc02-RS cells didn’t migrate faster but actually migrated slower by almost 50% as shown in Fig. 6.

**Fig. 7:**
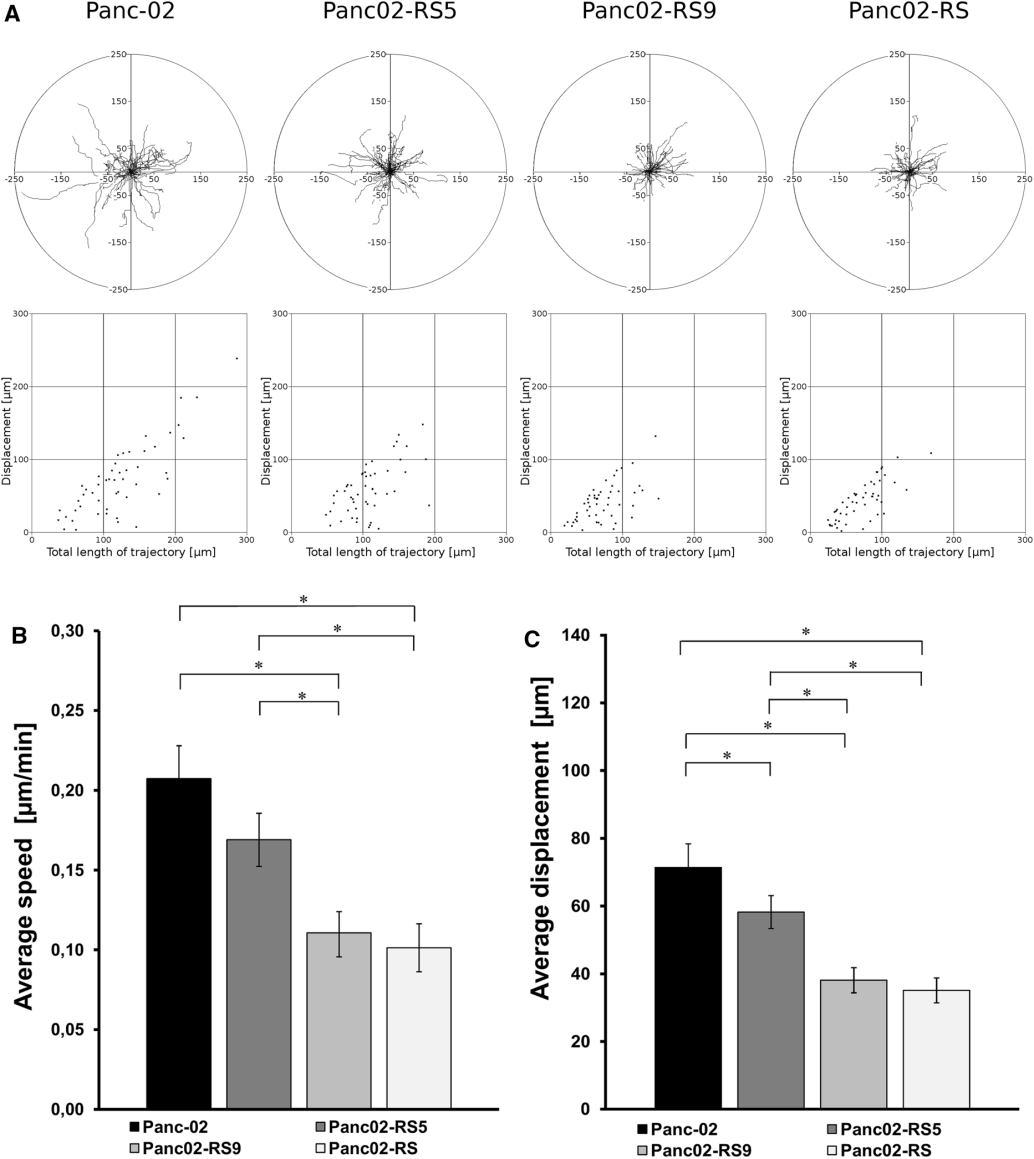
Cell motility of Panc-02 and Panc02-RS cell lines across different selection cycles. **a** 8 h time lapse video microscopy observation. **b** Graph showing average displacement through successive rounds of selection starting from Panc-02 wild type, continued with selection cycles 5 and 9 and finishing with Panc02-RS cell line, * p < 0.05. **c** Graph showing a speed of migration through successive rounds of selection starting from Panc-02 wild type, continued with selection cycles 5 and 9 and finishing with Panc02-RS cell line, * p < 0.05

During the selection process after cycles: 5 and 9 we preserved consecutive cell subpopulations. In Fig. 7b we see that subsequent cycles clearly lowered the speed of migration.

Each selection cycle decreased an average displacement and between cycles 5 and 9 we observed a sudden decrease in cell motility. IVMS proved it self to be a procedure giving very stable increase in the transition towards EMT.

Our expectation to observe higher migratory potential in Panc02-RS cells comparing to Panc-02 cells has not been fulfilled. We thought that may be the in vivo observation comes from the ease of Panc02-RS cell line to enter blood stream by migrating through the blood vessel wall. We tested this hypothesis using cell transmigration assay in a transwell.

**Fig. 8:**
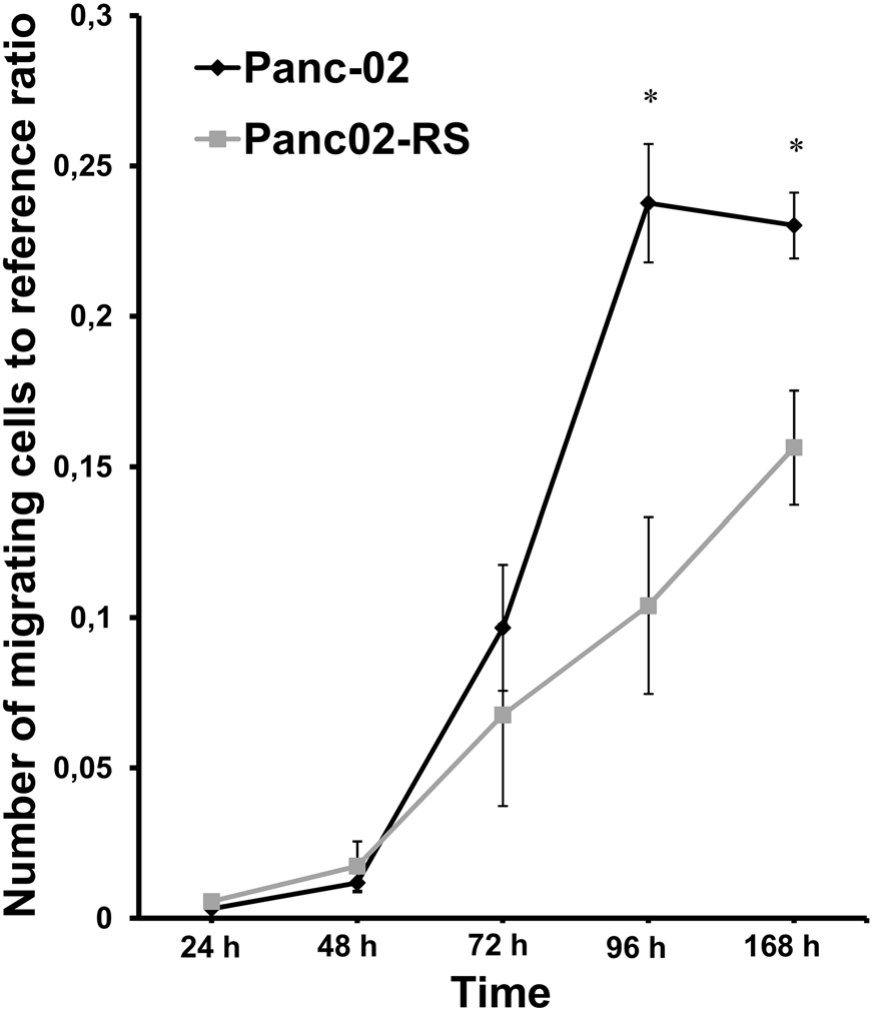
Invasive properties of Panc-02 and Panc02-RS cells on transwell invasion potential assay. Graph comparing the transmigrational potential of Panc-02 and Panc02-RS cell lines within 5 days time frame,* p < 0.05

Again a transwell test surprisingly showed that Panc-02 cell line has a greater potential for transmigration than Panc02-RS cell line as shown in Fig. 8. It is a visible trend but it has become clear after 96 h and 168 h that significantly more Panc-02 cells migrated through the membrane compared to Panc02-RS. It means that IVMS decreased the transmigrational potential. This observation indicates that nanomechanical elasticity is not a pre-requisite for the metastatic behavior of pancreatic cancer cells. In case of transmigration we need to keep in mind that it is not only a question of cell motility but also the stiffness of cells that have to squeeze to pass through the membrane (Nguyen et al. 2016). Note that spindle shape of Panc02-RS cells does not correlate with the transmigration potential in vitro but was already shown to correlate with the invasive potential in vivo.

### Molecular aspects of the observed processes

Until this moment we concentrated on the functional aspects of the investigated process. Everything we have seen so far has to be reflected by the molecular mechanism. As described above, IVMS led to the pre-selection of spindle-shaped Panc-02 cells, therefore we asked whether the microevolution of their metastatic sub-populations is related to EMT. EMT is a physiological process that plays an important role in embriogenesis and wound healing (Barriere et al. 2015). It is one of the decisive steps during the metastatic cascade of pancreatic cancer. We have observed slightly decreased E-cadherin and slightly increased N-cadherin levels were seen in Panc02-RS cells, when compared to wild type Panc-02 cells (data not shown). A “cadherin shift” i.e. a decrease of E-cadherin and an increase of N-cadherin expression is one of the hallmarks of EMT, therefore these observations may illustrate the involvement of EMT in Panc-02 heterogeneity (Nakajima et al. 2004).

**Fig. 9:**
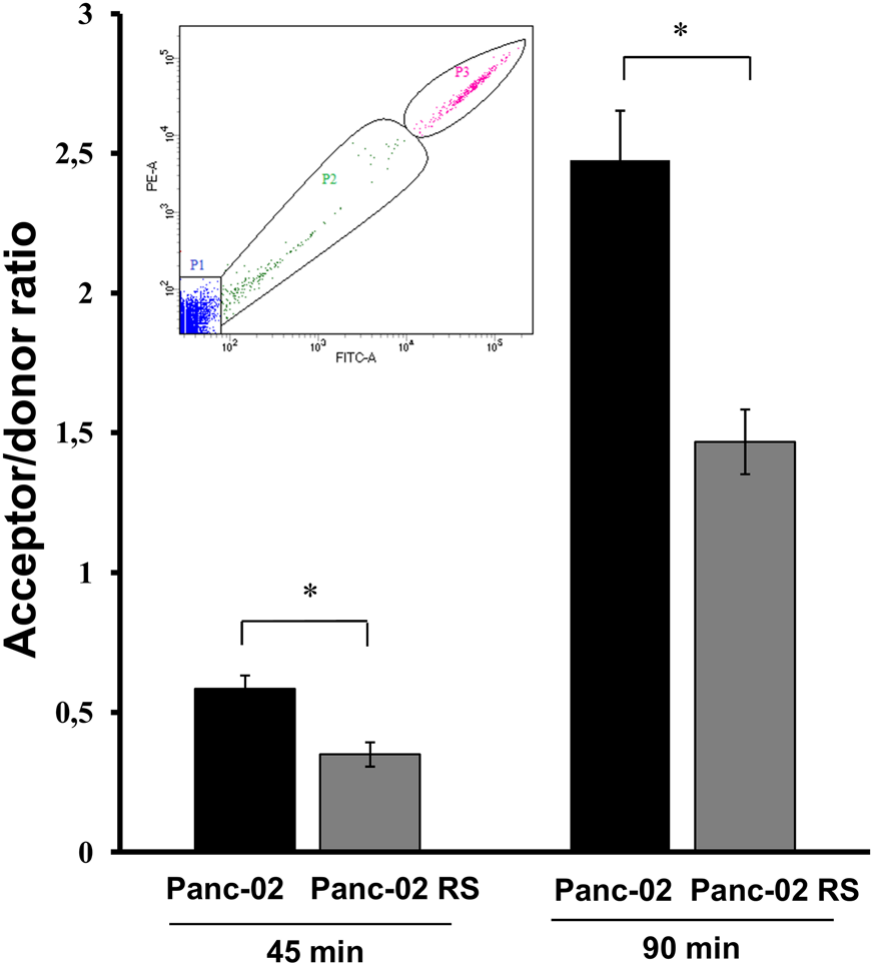
Gap junction intracellular communication (GJIC) in Panc-02 and Panc02-RS cell populations analyzed with flow cytometry. Upper left corner shows the gates used for flowcytometry measurements: P1: acceptor cells; P2: stain accepting cells; P3: donor cells. Graph shows an acceptor/donor ratio. Panc02-RS have shown a significant decrease in cell communication comparing to Panc-02 after 45 and 90 min, * p < 0.05

Following these results we decided to investigate the direct cell to cell communication. Neighboring cells can communicate through gap junctions that connect the cells interiors. This mechanism can be investigated as a passive transport of calceine from a donor to an acceptor cell and a result is presented as a ratio of a number of acceptor cells to the donor cells on a time dependent scale, see Fig. 9.

We have shown that Panc02-RS cell line has a decreased communication level comparing to Panc-02. It seems that Panc02-RS has less contacts with neighboring cells what fits to the previous result showing less vinculin in Panc02-RS cell line. On the other hand, Panc02-RS cells were characterized by a lower intensity of GJIC [gap junction intercellular communication] that their wild type counterparts (Fig. 9a), GJIC is a passive intercellular transfer of small metabolites (Holder et al. 1993). Cells well attached to the surface create more contact points with other cells. Panc-02 wild type cells have a stronger bond with the surface compared to the Panc02-RS cells. Since the cell division causes cells to partially detach from the surface and become more round this result complies with the growth curve in Fig. 3. EMT is often correlated with the Cx43 up-regulation and GJIC induction in cancer cell populations, therefore this observation may indicate a type III (incomplete) EMT (Kalluri and Weinberg 2009). We did not observe any differences in Snail-1 and Cx43 expression levels between Panc-02 and Panc02-RS cells (data not shown). Concomitantly, vinculin was preferentially recruited into focal contacts of Panc-02 cells, whereas a more diffuse distribution was seen in Panc02-RS cells. Most importantly, Panc02-RS cells displayed a considerably lower (50%) motile activity than their wild type counterparts (Figs. 6 and 7). These data stay in contrast with the outcome of in vivo experiment showing relatively high metastatic potential. Because the involvement of EMT in cancer progression is related to the induction of cancer cell motility, these data indicate that neither EMT nor cell motility account for relatively high metastatic potential of Panc-02 cells. One of the hallmarks of EMT is a decrease E-cadherin and an increase N-cadherin which we observed together with a decrease in the vinculin expression (data not shown). Immunocytofluorescence is not a quantitative method but qualitatively showed a trend towards EMT (data not shown).

These data show that the formation of pancreatic cancer invasive front is governed by paracrine signaling that affects cell invasiveness in a manner independent of cell motility. They also demonstrate the suitability of the novel IVMS assay for in vitro modeling of pancreatic cancer progression and passive shedding hypothesis investigation.

## Conclusions

**Fig. 10:**
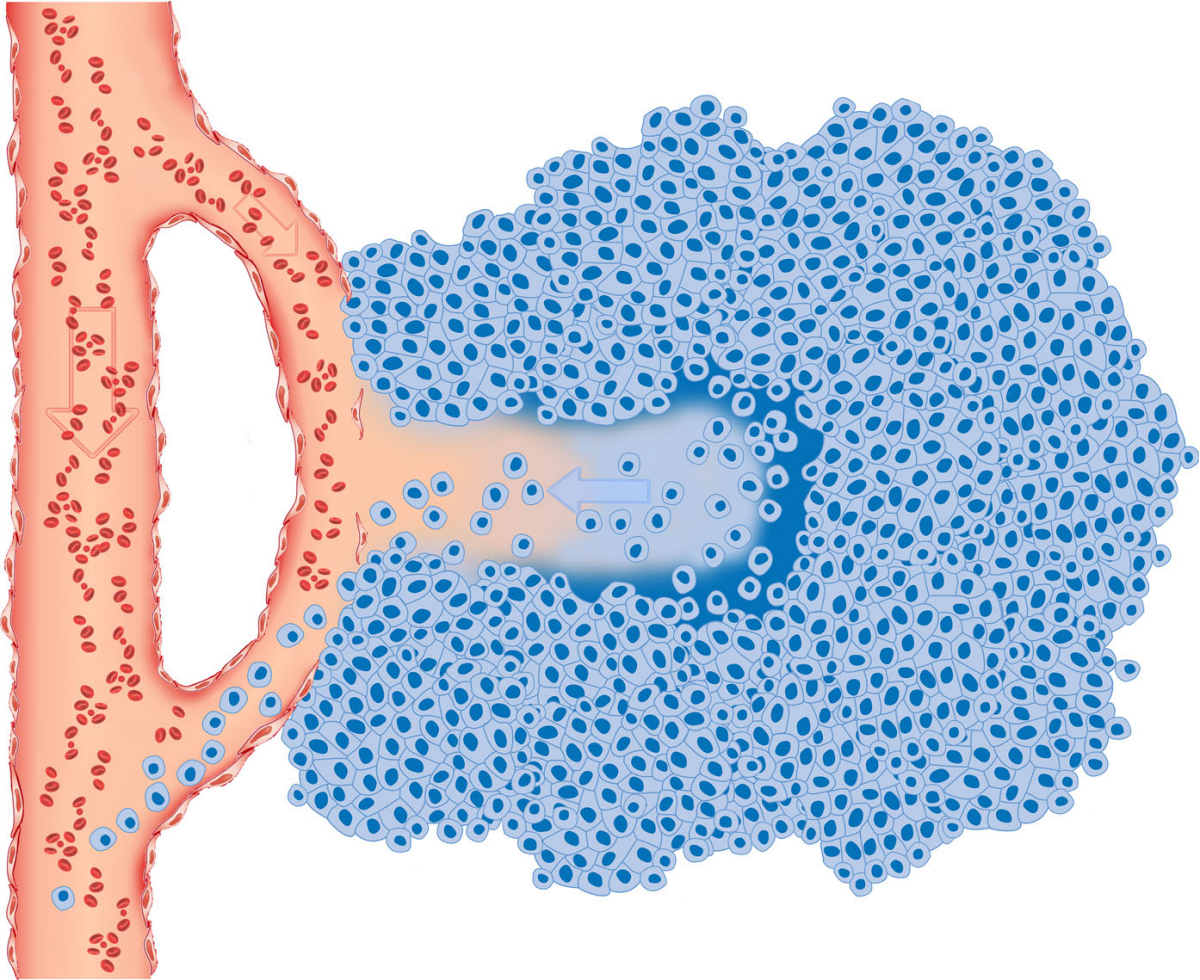
Passive shedding as an open gate for metastatic cells. Tumor growth requires blood supply. Blood vessels grow into tumor or existing blood vessels can be overtaken by growing tumor. Eventually those vessels become leaky due to apoptosis in hypoxic environment as well as due to tumor growth and mechanical distending. According to the suggested mechanism of passive shedding in the same hostile environment, with little oxygen, insufficient nutrient supply and constantly growing cell populations, cancer cells instead of apoptosis choose a sort of anergy when they roundup and detach from the stratum. If this mechanism will occur in a close proximity to the leaky blood vessel it becomes a way to passively migrate out of the tumor. Additionally this process is supported by the increased hydrostatic pressure from inside to outside of the tumor that can push metastatic cancer cells out into the blood stream

Our results support the hypothesis of passive shedding suggested by Munn et al. (Bockhorn et al. 2007). We have shown that despite much lower motility Panc02-RS cells had much greater metastatic potential in vivo comparing to wild type Panc-02 cells. We conclude that passive shedding may be a second path to metastasis and that the paradigm of metastasis should be broadend and include also passive shedding mechanisms as proposed in Fig. 10.

Espetially for long distances hematogenous dissemination (Wong and Hynes 2006) where blood flow play a crutial role, passive shedding can explain the biological processes behind the fact that 3–4 × 10^6^ malignant cells/day per gram of the tumor enter into the blood stream (Butler and Gullino 1975). Passive shedding can be described as a passive process where cell enters into a anabiosis (caused by hypoxia, acidity, tumor overgrowth, lack of nutrients etc.). In our experiments we observed growing number of living cells in suspension with increased cell culture density. Those cells were resistant to apoptosis caused by cell detachment. Unlimited tumor growth leads to high density of cancer cells and pathological tumor vascularisation. Tumor vasculature is unable to “feed” all cancer cells and similar things happend to cells observed in the lab, namely overgrowth of the tumor colony followed by an increased number of suspended cells. Pathological vasculature results in the vessels discontinuity and leakage. Additionally increased apoptosis and necrosis of endothelial cells enlarges the gaps in blood vessels. Panc-02 is known for its blood vessels leakiness (Krzykawski et al. 2015).

Our preliminary experiments on other cell lines and produced similar results. We decided to broaden the spectrum of experiments on single cell line rather that testing multiple cell lines. We saw it as more important to have deeper understanding of the process in stead of proving the very basic experiments on the single cell line. Eventually these results will have to be repeated and broaden by independent laboratories.

We have developed IVMS as a new technique imitating metastasis. IVMS increases metastatic potential of a pancreatic cancer cell line, Panc-02. This way we omit evolutionary pressure of the immune system using only simple laboratory techniques and concentrate on the cellular processes. In vivo the pathophysiological influence of immune system is extremely important but in this study we decided to concentrate on the creation of a research model for passive shedding. During the selection, immune system would force additional selective pressure.

So lets connect the facts. We propose that due to high cell density many cancer cells enter into freely floating state. At the same time blood vessels flushing the tumor become leaky. It makes an open gate for freely floating metastatic cancer cells to shed into the blood stream as shown in Fig. 10. There is a gap in understanding how cells that clearly resemble low metastatic capabilities in vitro, seem to be more metastatic in vivo. Our results fill the gap and show that increased metastatic potential can corelate with decreased motility as long as it correlates with resistance to transient detachment. This way large number of cells can enter blood vessels and some of them will metastasise to distant organs. Additionally intratumoral pressure is a force that will push metastatic freely floating cells into the blood stream (Pesonen et al. 2011). IVMS is a technique facilitating the observation of passive shedding by increasing number of cells in suspension.

Passive shedding hypothesis may be usefull in searching for new diagnostic and therapeutic markers. In clinical conditions an observation of suspended cancer cells in a blood stream may be a significant information to determine the stage of tumor development (Wong and Hynes 2006). Detached cells may express different surface markers that could be usefull to determine cancer virulence but may also be used as therapeutic markers.
